# Sequence variants of interleukin 6 (*IL-6*) are significantly associated with a decreased risk of late-onset Alzheimer's disease

**DOI:** 10.1186/1742-2094-9-21

**Published:** 2012-01-24

**Authors:** Shih-Yuan Chen, Ta-Fu Chen, Liang-Chuan Lai, Jen-Hau Chen, Yu Sun, Li-Li Wen, Ping-Keung Yip, Yi-Min Chu, Yen-Ching Chen

**Affiliations:** 1 Institute of Epidemiology and Preventive Medicine, College of Public Health, National Taiwan University, Taipei, Taiwan; 2 Department of Neurology, National Taiwan University Hospital, Taipei, Taiwan; 3 Graduate Institute of Physiology, College of Medicine, National Taiwan University, Taipei, Taiwan; 4 Department of Geriatrics and Gerontology, National Taiwan University Hospital, Taipei, Taiwan; 5 Department of Neurology, En Chu Kong Hospital, Taipei, Taiwan; 6 Center of Laboratory Medicine, En Chu Kong Hospital, Taipei, Taiwan; 7 Center of Neurological Medicine, Cardinal Tien Hospital,Taipei, Taiwan; 8 Department of Laboratory Medicine, Cardinal Tien Hospital,Taipei, Taiwan; 9 Department of Medicine, School of Medicine, Fu-Jen Catholic University, Taipei, Taiwan; 10 Department of Public Health, National Taiwan University, Taipei, Taiwan; 11 Research Center for Genes, Environment and Human Health, College of Public Health, National Taiwan University, Taipei, Taiwan

**Keywords:** *IL-6*, SNP, Haplotype, Alzheimer's disease, Inflammation

## Abstract

**Background:**

Interleukin 6 (IL-6) has been related to beta-amyloid aggregation and the appearance of hyperphosphorylated tau in Alzheimer's disease (AD) brain. However, previous studies relating *IL-6 *genetic polymorphisms to AD included few and unrepresentative single nucleotide polymorphisms (SNPs) and the results were inconsistent.

**Methods:**

This is a case-control study. A total of 266 patients with AD, aged≧65, were recruited from three hospitals in Taiwan (2007-2010). Controls (n = 444) were recruited from routine health checkups and volunteers of the hospital during the same period of time. Three common *IL-6 *haplotype-tagging SNPs were selected to assess the association between *IL-6 *polymorphisms and the risk of late-onset AD (LOAD).

**Results:**

Variant carriers of *IL-6 *rs1800796 and rs1524107 were significantly associated with a reduced risk of LOAD [(GG + GC vs. CC): adjusted odds ratio (AOR) = 0.64 and (CC + CT vs. TT): AOR = 0.60, respectively]. Haplotype CAT was associated with a decreased risk of LOAD (0 and 1 copy vs. 2 copies: AOR = 0.65, 95% CI = 0.44-0.95). These associations remained significant in *ApoE e4 *non-carriers only. Hypertension significantly modified the association between rs2069837 polymorphisms and the risk of LOAD (*p*_interaction _= 0.03).

**Conclusions:**

*IL-6 *polymorphisms are associated with reduced risk of LOAD, especially in *ApoE e4 *non-carriers. This study identified genetic markers for predicting LOAD in *ApoE e4 *non-carriers.

## Background

Dementia is a neurodegenerative disease characterized by decline or loss in cognitive function. Alzheimer's disease (AD) is the most common cause of dementia. In the United States in 2006, AD was the fifth leading cause of death in the elderly (age 65 or older) [1]. In Taiwan, the prevalence of dementia is around 1.7-4.3% among the elderly [2] and the number of dementia patients keeps increasing. Therefore, dementia has become an important health issue in aging populations.

Interleukin-6 (IL-6), an inflammatory cytokine, plays an important role in the development and differentiation of neurons in both peripheral and central nervous system [3]. IL-6 promotes the activation of microglia [4] and then induces the synthesis of acute phase proteins [5] and phosphorylation of tau protein in neurons [6]. In AD brain, microglia and astrocytes are stimulated by IL-6 and are recruited to release proinflammatory cytokines and acute-phase proteins, such as C-reactive protein (CRP) [7]. Therefore, IL-6 plays a pivotal role in brain inflammation that maybe important in AD pathogenesis.

Previous studies relating *IL-6 *polymorphisms to AD risk have been inconsistent. A Caucasian study found that the CC genotype of *IL-6 *promoter SNP rs1800795 was significantly associated with an increased risk for AD [8]. However, this association has not been observed in other Caucasian studies [9-11]. In addition, the GG genotype of promoter SNP rs1800796 has been associated with a decreased risk for AD in two Chinese populations [12,13]. For the latter study [13], a significant association was observed only for *Apolipoprotein E *(*ApoE*) *e4 *carriers. In contrast, no significant association was observed in a Japanese population [14]. Furthermore, the variant rs13447446 was very rare in a Korean study [minor allele frequency (MAF) = 0.006] [15] and a Chinese study (MAF = 0) [12] and thus no association analysis was performed. In summary, previous studies (see Table 1) have been inconsistent in relating *IL-6 *polymorphisms to AD risk. This may be attributable to different ethnic groups, SNPs selected, study period, or sample size.

**Table 1:** Previous studies relating *IL-6 *polymorphisms to AD risk

Reference	Population (AD:control)	SNP (w.t./variant)^a^ Haplotype (SNP)	Results	Limitations
Arosio et al. Neurobiol. Aging 2004, 25:1009-15 [9]	Italian (65:65)	rs1800795 (G/C)	No significant association	◈Italian only ◈One SNP only ◈Small sample size
Licastro et al. Neurobiol. Aging 2003, 24:921-26 [8]	Italian (332:393)	**rs1800795 (G/C)**	rs1800795: CC vs. GG: OR = 1.62, 95% CI = 1.20-2.17	◈Italian only ◈One SNP only
Depboylu et al. Geriatr. Cogn. Disord 2004, 17:170-73 [10]	German (113:108)	rs1800795 (G/C)	No significant association	◈German only ◈One SNP only
van Oijen et al. Neurosci. Lett. 2006, 402:113-17 [11]	Netherlander (483:5636)	rs1800795 (G/C)	No significant association	◈Netherlanders only ◈One SNP only
He et al. Neurol. Sci. 2010, 31:165-8 [12]	Chinese (318:324)	**rs1800796 (C/G)** rs13447446 (G/C)	rs1800796: GG vs. CC: OR = 0.42, 95% CI = 0.20-0.89	◈Chinese only ◈Not applicable for rs13447446 (No GC or CC variants)
Wang et al. Neurosci. Lett. 2010, 482:260-3 [13]	Chinese (341:421)	**rs1800796 (C/G)** rs7802308 (A/T) **haplotype rs1800796/rs7802308**	rs1800796 (in ApoE *e4 *carrier): 1) CC vs. CG + GG: OR = 3.3, 95% CI = 1.64-6.67 2) A risk haplotype (CA) (OR = 2.24) 3) A protective haplotype (GA) (OR = 0.41)	◈Chinese only ◈Significant only in *ApoE e4-*carrier ◈rs7802308 is a rare SNP for analysis
Nishimura et al. Neurosci. Lett. 2004, 368:140-3 [14]	Japanese (172:163)	rs1800796 (C/G)	No significant association	◈Japanese only ◈One SNP only

^a ^Words underlined indicates SNPs genotyped in our study.

*Abbreviations*: OR, odds ratio; AD, Alzheimer's disease; SNP, single nucleotide polymorphism; w.t., wild type.

IL-6 plays an important role in inflammation. Past studies relating *IL-6 *polymorphisms to AD risk have been limited to few and unrepresentative SNPs in *IL-6*. Some SNPs (e.g., rs13447446) even showed no variations in Asians. Therefore, this study used a systematic approach to select haplotype-tagging SNPs (htSNPs) in *IL-6 *to explore their association with AD risk. Because *ApoE e4 *status and some vascular risk factors [e.g., hypertension, hyperlipidemia, and type 2 diabetes mellitus (DM)] may affect the pathogenesis of dementia [16-18], this study further explored the effect of modification by these factors.

## Methods

### Study population

This was a case-control study. A total of 294 late-onset AD (LOAD) cases were recruited from the neurology clinics of three teaching hospitals in northern Taiwan from 2007 to 2010. Healthy controls (n = 503) were recruited from elderly health checkup and volunteers of the hospital during the same period of time. All participants were Taiwanese aged 65 years or older. Participants with a history of depression, dementia subtypes other than AD, Parkinson's disease, hemorrhagic stroke, cerebral infarction, or organic brain tumors were excluded. After further exclusion of participants without blood samples, a total of 266 LOAD and 444 controls were included for data analyses. The study protocol has been approved by the Institutional Review Boards of National Taiwan University Hospital, En Chu Kong Hospital, and Cardinal Tien's Hospital. Written informed consent was obtained from each study participant. The consent from the legal guardian/next of kin was obtained when patients had serious cognitive impairment. A self-reported questionnaire was administered to collect information on demography, comorbidity (e.g., hypertension, hyperlipidemia, and type 2 DM), and lifestyle.

### Dementia evaluation

At each hospital, potential dementia cases were diagnosed by a neurologist. The Mini-Mental State Examination (MMSE) was used to evaluate participants with cognitive impairment. The diagnosis of probable dementia was evaluated using Diagnostic and Statistical Manual of Mental Disorders (Fourth Edition) [19] criteria. Head magnetic resonance imaging and computed tomography were taken to exclude participants with organic lesions. National Institute of Neurological and Communicative Disorders and Stroke and the Alzheimer's Disease and Related Disorders Association (NINCDS-ADRDA) Alzheimer's Criteria [20] was used to diagnose probable AD. The cognitive function of controls was assessed by using Short Portable Mental Status Questionnaire [21] to exclude participants with possible dementia and other mental disorders.

### SNP selection and genotyping assay

Genotype data of common (frequency ≥ 5%) *IL-6 *SNPs were downloaded from the International HapMap Project (http://hapmap.ncbi.nlm.nih.gov) for Han Chinese in Beijing, China (CHB). Haploview (http://www.broadinstitute.org/haploview/haploview) was used to define haplotype block by using the modified Gabriel algorithm [22,23]. Two htSNPs were selected among four common SNPs using tagSNP program [24]. For comparison purpose, SNP rs1800796 was further included because it has been related to AD risk [12,13].

Blood samples were collected in tubes containing sodium EDTA from each participant for genotyping. After centrifugation, genomic DNA was extracted from buffy coats using a QuickGene-Mini80 system (Fujifilm, Tokyo, Japan) and then stored in a -80°C freezer. Genotypes of *ApoE *SNPs were determined using the assay developed by Chapman et al. [25]. Genotypes of *IL-6 *htSNPs were determined by Taqman Assay (Applied Biosystems Inc., CA, USA) with genotyping success rate greater than 95%. Five percent of internal samples were selected and replicated for quality control, and the concordance rate was 100%.

### Statistical analysis

The distributions of demographic characteristics between cases and controls were compared using Student's *t*-test for normally distributed continuous variables and a chi-square test for categorical variables. The Hardy-Weinberg equilibrium (HWE) test was performed among controls to examine possible genotyping error and selection bias for each SNP. The *ApoE *diplotypes (*e2/e2, e2/e3, e3/e3, e2/e4, e3/e4*, and *e4/e4*) were determined by *ApoE*112 (*rs429358*) and *ApoE*158 (*rs7412*) [26]. *ApoE e4 *carriers were defined as participants carrying *e2/e4, e3/e4*, or *e4/e4 *diplotypes; participants carrying other diplotypes (*e2/e2, e2/e3*, and *e3/e3*), were defined as *ApoE e4 *non-carriers. The expectation-maximization algorithm was utilized to estimate haplotype frequencies. Logistic regression models were performed to estimate SNP- and haplotype-specific odds ratios (OR) and 95% confidence intervals (CI) for LOAD adjusting for age, gender, education, and *ApoE e4 *status. Type I errors were controlled by using false discovery rate (FDR) [27].

A likelihood ratio test was used to evaluate how *ApoE e4 *status and vascular risk factors (e.g., type 2 DM, hypertension, and hyperlipidemia) modified the association between *IL-6 *polymorphisms and risk of LOAD. Stratified analyses were performed subsequently to assess the association between *IL-6 *polymorphisms and risk of LOAD by these vascular risk factors. SAS version 9.2 (SAS Institute, Cary, NC) was used for statistical analyses and all statistical tests were two-sided.

## Results

### Characteristics of the study population

This study included 266 incident LOAD cases and 444 controls. Compared with controls, LOAD cases were older (79.8 vs. 73.1 years old), included more females (64% vs. 52%), had a lower education level (≤ 6 years: 50% vs. 11%), and included fewer with hypertension history (38% vs. 54%) or hyperlipidemia (18% vs. 30%), and more *ApoE e4 *carriers (39% vs. 15%, Table 2). The distributions of body mass index (BMI), cigarette smoking, alcohol consumption, and type 2 DM were similar between LOAD cases and controls.

**Table 2:** Characteristics of the study population

Variables	AD N = 266	Control N = 444
Age (mean ± SD)	79.8 ± 7.9*	73.1 ± 7.1
Female (%)	170 (64)*	233 (52)
Education (%)		
≦6 years	134 (50)*	54 (11)
6-12 years	90 (34)	177 (40)
> 12 years	39 (15)	214 (48)
BMI at age 40s, kg/m^2^(mean ± SD)	22.6 ± 3.1	22.4 ± 2.8
Cigarette smoking (%)	62 (23)	77 (17)
Alcohol consumption (%)	32 (12)	50 (11)
Type 2 DM (%)	48 (17)	62 (13)
Hypertension (%)	101 (38)*	239 (54)
Hyperlipidemia (%)	49 (18)*	135 (30)
*ApoE e4 *carriers (%)	104 (39)*	66 (15)

* *p *value < 0.05 was obtained by comparing AD and controls.

*Abbreviations*: AD, Alzheimer's disease; BMI, body mass index; DM, diabetes mellitus; *ApoE e4*, apolipoprotein E *e*4.

### *ApoE *SNPs

Neither of the two *ApoE *SNPs (rs429358 and rs7412) was out of HWE (Table 3). *AopE *rs429358 (*ApoE*112) is a common SNP in this Chinese population (MAF = 0.08 in controls) and in Caucasians (MAF = 0.15, dbSNP dataset). In contrast, HapMap data showed that rs429358 is a rare variant in Chinese (CHB, MAF = 0) and Japanese (JPN, MAF = 0.01). The MAF is similar for *ApoE *rs7412 (*ApoE*158) across ethnic groups: Chinese (HapMap CHB: 0.11; controls of this study: 0.08), Caucasian (dbSNP: 0.08), and Japanese (HapMap: 0.05).

**Table 3:** Characteristics of *IL-6 *haplotype-tagging SNPs and *ApoE *SNPs

				HapMap or dbSNP			This study					
SNP name	Nucleotide change	Location	**rs no**.	MAF			Controls			Cases		
			
				CEU	JPT	CHB	MAF	HWE		MAF	HWE	
										
								χ^2^	*p*		χ^2^	*p*
SNP1	C→G	Promoter	rs1800796	0.04	0.14	0.23	0.26	0.98	0.32	0.22	0.36	0.55
SNP2	A→G	Intron	rs2069837	0.08	0.10	0.17	0.19	2.59	0.11	0.16	0.04	0.84
SNP3	T→C	Intron	rs1524107	0.05	0.18	0.29	0.27	1.31	0.25	0.22	0.22	0.64
*ApoE*112	T→C	Exon	rs429358	0.15*	0.01	0.00	0.08	0.17	0.68	0.23	1.24	0.27
*ApoE*158	C→T	Exon	rs7412	0.08*	0.05	0.11	0.08	1.49	0.22	0.06	0.001	0.97

*Abbreviations*: CEU, Utah residents with Northern and Western European ancestry from the CEPH collection; JPT, Japanese in Tokyo, Japan; CHB, Han Chinese in Beijing; HWE *p, p *value for Hardy-Weinberg equilibrium test; MAF, minor allele frequency; SNP, single nucleotide polymorphism.

The degree of freedom is 1 for all HWE tests.

* MAF data were obtained from dbSNP because the data was not available from the HapMap Project.

### *IL-6 *SNPs and AD risk

Three *IL-6 *SNPs (rs1800796, rs2069837, and rs1524107) were genotyped. The MAFs of these SNPs among controls ranged from 0.19 to 0.27, which were similar to the MAFs (0.20-0.25) of CHB genotype data from the International HapMap Project (Table 3). In contrast, MAF of these three IL-6 SNPs were lower in Japanese (0.12, 0.10 and 0.18) and in Caucasians (0.04, 0.08, and 0.05) based on HapMap data. All *IL-6 *SNPs were in HWE among controls. For each SNP, the genotype frequencies were similar between cases and controls (data not shown).

Participants carrying one or two copies of variant SNP1 (rs1800796) or SNP3 (rs1524107) had a significantly decreased risk of LOAD [SNP1: adjusted OR (AOR) = 0.64, 95% CI = 0.43-0.94; SNP3: AOR = 0.60, 95% CI = 0.41-0.89] compared with those carrying 0 copies. These results remained significant after controlling for FDR under dominant or additive model (Table 4). After stratification by *ApoE e4 *status, SNP1 and SNP3 were significantly associated with decreased LOAD risk among *ApoE e4 *non-carriers (SNP1: AOR = 0.56, 95% CI = 0.35-0.90; SNP3: AOR = 0.55, 95% CI = 0.35-0.88, Table 4). These results remained statistically significant after controlling for FDR (Table 4). In contrast, results were not significant among *ApoE e4 *carriers.

**Table 4:** Association between *IL-6 *SNPs and LOAD risk by *ApoE e4 *status

**Dominant model**						**Additive model**	
	
	**0 copies**		**1 or 2 copies**				
			
	**Case/** **control**	**AOR**	**Case/** **control**	**AOR (95% CI)**	***p***	**AOR (95% CI)**	***p***
	
**SNP1**	162/245	1.00	104/196	0.64 (0.43-0.94)*	**0.02**	0.67 (0.50-0.91)*	**0.01**
			
*ApoE e4 *non-carriers	100/207	1.00	61/166	0.56 (0.35-0.90)*	**0.02**	0.61 (0.42-0.90)*	**0.01**
*ApoE e4 *carriers	61/37	1.00	43/29	0.82 (0.40-1.70)	0.60	0.81 (0.49-1.34)	0.28
**SNP2**	180/293	1.00	75/141	0.78 (0.51-1.19)	0.25	0.80 (0.56-1.14)	0.21
			
*ApoE e4 *non-carriers	114/249	1.00	40/118	0.72 (0.43-1.20)	0.21	0.72 (0.46-1.12)	0.14
*ApoE e4 *carriers	66/43	1.00	35/22	0.89 (0.42-1.91)	0.77	0.99 (0.54-1.83)	0.59
**SNP3**	163/242	1.00	102/199	0.60 (0.41-0.89)*	**0.01**	0.66 (0.49-0.90)*	**< 0.01**
			
*ApoE e4 *non-carriers	101/204	1.00	60/169	0.55 (0.35-0.88)*	**0.01**	0.60 (0.41-0.89)*	**0.01**
*ApoE e4 *carriers	61/37	1.00	42/29	0.73 (0.36-1.52)	0.40	0.81 (0.48-1.34)	0.23

All models were adjusted for age, gender, education, and *ApoE e4 *status.

*Abbreviations*: AOR, adjusted odds ratio; LOAD, late-onset Alzheimer's disease; SNP, single nucleotide polymorphism.

* *p *value remained significant after controlling for type I error by using FDR.

### *IL-6 *haplotypes and AD risk

**Figure 1:**
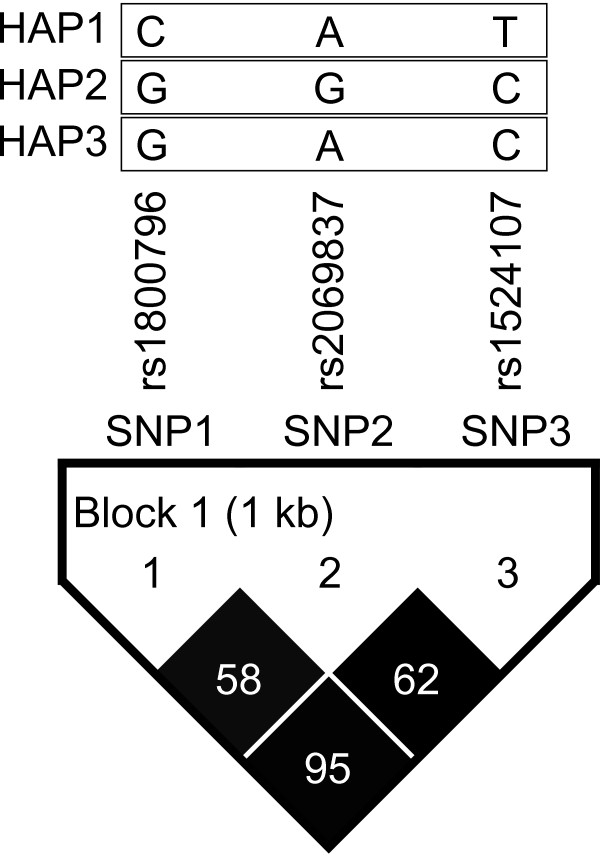
***IL-6 *linkage disequilibrium (LD) plot**. This plot was generated by using a Haploview program. Three SNPs form one block. The SNP name, e.g., SNP1, SNP2, and SNP3; indicates the three htSNPs genotyped in this study. Three common haplotypes (frequency ≥ 0.05) were identified. The level of pair wise r^2^, which indicates the association degree between two SNPs in LD block, is shown numerically in the cell of the LD structure. The level of pair-wise D', which indicates the strength of LD between two SNPs, is shown in the LD structure in gray scale.

Three common (frequency ≥ 5%) htSNPs spanning *IL-6 *formed one haplotype block, which was determined by the modified Gabriel et al. algorithm [22,23] (Figure 1). Three common haplotypes (CAT, GGC, and GAC) with a cumulative frequency of 99% in controls were identified in *IL-6 *(Table 5). Because HAP1 CAT is a "major" haplotype (haplotype frequency > 0.50), 2 copies of HAP1 were used as the reference group to be consistent with the reference groups used for HAP2 and HAP3, which are "minor" haplotypes (haplotype frequency < 0.50). Participants carrying 0 or 1 copies of major HAP1 CAT had a decreased risk of LOAD (AOR = 0.65, 95% CI = 0.44-0.95) compared to those carrying 2 copies of major HAP1.

**Table 5:** Association between *IL-6 *haplotypes and LOAD

	Prevalence in controls, %	2 copies		1 or 0 copies		
			
		Case/Control	AOR	Case/Control	AOR (95% CI)	*p*
HAP1 (CAT)^a^	73.3	159/244	1.00	107/200	**0.65 (0.44-0.95)**	**0.02**
*ApoE e4 *non-carriers		99/206	1.00	62/170	**0.57 (0.36-0.91)**	**0.02**
*ApoE e4 *carriers		59/37	1.00	45/29	0.85 (0.42-1.76)	0.67

	Prevalence in controls, %	0 copies		1 or 2 copies		
			
		Case/Control	AOR	Case/Control	AOR (95% CI)	*p*

HAP2 (GGC)	17.8	263/439	1.00	3/5	1.10 (0.16-7.58)	0.92
HAP3 (GAC)	8.1	260/442	1.00	6/2	2.56 (0.39-16.95)	0.33

^a ^Two copies of HAP1 was used as the reference group because it is a "major" haplotype (haplotype frequency > 0.5).

*Abbreviations*: AOR, adjusted odds ratio; HAP, haplotype; LOAD, late-onset Alzheimer's disease.

All models were adjusted for age, gender, education, and *ApoE e4 *status.

### Effect modification by vascular risk factors

Among the vascular risk factors explored in this study (e.g., hypertension, type 2 DM, and hyperlipidemia), hypertension was the only factor that significantly modified the association between *IL-6 *SNPs and risk of LOAD (SNP2: *p*_interaction _= 0.03 under dominant model, Table 6). Hypertension history significantly decreased the risk of LOAD (AOR = 0.41, 95% CI = 0.28-0.60, Table 6). After stratification by hypertension status, hypertensive patients carrying variant SNP2 had a decreased risk of LOAD (AOR = 0.43, 95% CI = 0.22-0.86, Table 6). Similar findings were observed for SNP1 and SNP3 (SNP1: AOR = 0.53, 95% CI = 0.29-0.96; SNP3: AOR = 0.53, 95% CI = 0.29-0.97). After stratification by type 2 DM, non-DM patients carrying variant SNP1 and SNP3 were associated with a decreased LOAD risk (AOR = 0.54 and 0.58, Table 6). The associations above remained statistically significant after controlling for FDR.

**Table 6:** Association between *IL-6 *SNPs and LOAD by hypertension or type 2 DM

Dominant model					*p*_interaction_^a^
	**0 copies**		**1 or 2 copies**		
			
	**Case/** **Control**	**AOR**	**Case/** **Control**	**AOR (95% CI)**	

Hypertension	266/443			**0.41 (0.28-0.60)**	NA
SNP1					
No	96/105	1.00	69/98	0.64 (0.37-1.09)	0.54
Yes	66/139	1.00	35/98	0.53 (0.29-0.96)*	
SNP2					
No	106/131	1.00	52/67	1.09 (0.61-1.92)	**0.03**
Yes	74/161	1.00	23/74	0.43 (0.22-0.86)*	
SNP3					
No	99/102	1.00	66/100	0.59 (0.34-1.01)	0.74
Yes	64/139	1.00	36/99	0.53 (0.29-0.97)*	

Type 2 DM	263/442			**0.47 (0.30-0.74)**	NA
SNP1					
No	136/210	1.00	81/167	0.58 (0.38-0.90)*	0.48
Yes	25/33	1.00	23/29	0.91 (0.36-1.31)	
SNP2					
No	146/251	1.00	61/119	0.77 (0.48-1.23)	0.96
Yes	33/40	1.00	14/22	0.78 (0.28-2.20)	
SNP3					
No	136/207	1.00	80/171	0.54 (0.35-0.84)*	0.39
Yes	26/33	1.00	22/28	0.92 (0.37-2.31)	

^a ^*p*_interaction _value was obtained by using the dominant model.

All models were adjusted for age, gender, education, and *ApoE e4 *status.

*Abbreviations*: AOR, adjusted odds ratio; DM, diabetes mellitus; LOAD, late-onset Alzheimer's disease; SNP, single nucleotide polymorphism; NA, not applicable.

* Result remained significant after controlling for type I error by using FDR.

For *IL-6 *haplotypes, vascular risk factors or *ApoE e4 *status did not significantly modify the association between *IL-6 *haplotypes and the risk of LOAD (*p*_interaction _> 0.05). After stratification by hypertension history, hypertensive patients carrying 0 or 1 copy of HAP1 had a decreased risk of LOAD (AOR = 0.52, 95% CI = 0.29-0.95, Table 7). After stratification by type 2 DM, non-DM patients carrying minor HAP1 were associated with a decreased LOAD risk (AOR = 0.59, 95% CI = 0.39-0.92, Table 7).

**Table 7:** Association between *IL-6 *haplotype CAT and LOAD by hypertension or type 2 DM

	2 copies^a^		0 or 1 copy		*p*_interaction_^b^
			
	Case/Control	AOR	Case/Control	AOR (95% CI)	
Hypertension					
No	94/104	1.00	71/100	0.67 (0.39-1.15)	0.49
Yes	65/139	1.00	36/100	**0.52 (0.29-0.95)**	
Type 2 DM					
No	133/209	1.00	84/171	**0.59 (0.39-0.92)**	0.36
Yes	25/33	1.00	23/29	0.91 (0.36-2.26)	

*Abbreviation*: AOR, adjusted odds ratio; DM, diabetes mellitus; LOAD, late-onset Alzheimer's disease.

All models were adjusted for age, gender, education, and *ApoE e4 *status.

^a ^2 copies of haplotype CAT was used as the reference group because it is a major haplotype.

^b ^*p*_interaction _value was obtained by using the dominant model.

## Discussion

In addition to replicating findings for rs1800796 explored in previous Asian studies [12-14], this study for the first time identifies two *IL-6 *htSNPs (rs2069837 and rs1524107) representative for the Chinese population, and rs1524107 showed a significant association with LOAD. We found that a variant of SNP rs1800796 is significantly associated with decreased risk of LOAD, which is consistent with the findings from a Chinese study [12]. In addition, we found that the intronic SNP rs1524107 and a haplotype CAT have a significant protective effect on LOAD risk, which has not been reported previously. Unlike rs1800796 and rs1524107, variant rs2068937 was not significantly associated with LOAD (Table 4). Although they are located in one block [i.e., highly linkage disequilibrium (LD) with dark gray in Figure 1], rs2068937 shows a low correlation with rs1800796 (r^2 ^= 0.58) and rs1524107 (r^2 ^= 0.62) which explains its lack of a significant association with LOAD.

**Figure 2:**
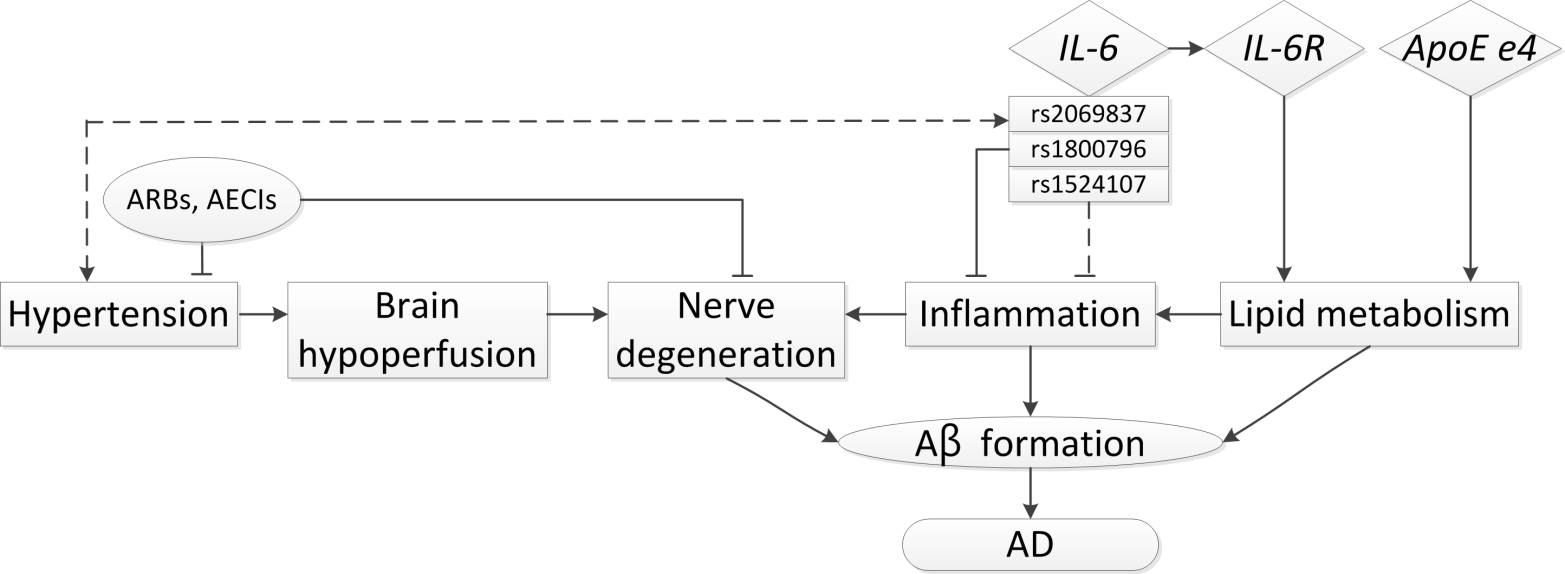
**Postulated pathway of *IL-6 *and factors involved in the pathogenesis of dementia**. Solid lines indicate pathways that have been well documented; dotted lines indicated speculative pathways. Abbreviations: Aβ, beta amyloid; IL-6R, interleukin 6 receptor; ARBs, angiotensin receptor blockers; AECIs, angiotensin-converting enzyme inhibitors; *ApoE e4*, apolipoprotein E e4; AD, Alzheimer's disease.

rs1800796 is a promoter SNP, which may enhance transcription efficiency [28] and affect plasma CRP levels [29] and subsequent inflammatory responses. These results indicate that the promoter SNP rs1800796 might be involved in the course of inflammation, and the variant rs1800796 could alter IL-6 production in the development of AD. Sequence variants of rs1524107 may affect disease risk via their effect on alternative splicing, e.g., altering mRNA folding or the stability of mRNA structure, and subsequent protein production. Besides, the most common *IL-6 *haplotype CAT, composed of three major alleles, was associated with decreased risk of LOAD. Therefore, carrying 0 or 1 copies of CAT showed consistent protective effects on LOAD risk, as did the variant *IL-6 *SNPs. These sequence changes may either block or attenuate inflammation signaling which leads to reduced risk of AD. This supports the finding of a protective effect from *IL-6 *polymorphisms on LOAD. The postulated mechanism of IL-6 in LOAD is demonstrated in Figure 2.

Although *ApoE e4 *status is a well-known predictor for AD risk, over half of AD cases (61% in this study, Table 2) do not carry the *ApoE e4 *allele. Therefore, prediction of LOAD risk among *ApoE e4 *non-carriers becomes an important task. This study found significant associations between rs1800796 or rs1524107 and LOAD in *ApoE e4 *non-carriers, which remained statistically significant after correction for type I error by using FDR. A Chinese study [13] found that the variant rs1800796 was associated with AD risk in *ApoE e4 *carriers but not in the wider population. This may be a chance finding because a significant protective effect of rs1800796 variants on AD risk was observed only after stratification by *ApoE e4 *status. In contrast, both He et al. [12] and our study found a significant association before stratification. A previous study showed that elevated serum IL-6 induces hypertriglyceridemia [30], which manifests before the deposition of beta amyloid (Aβ) in AD [31]. Therefore, *IL-6 *polymorphisms may be important in predicting LOAD in *ApoE e4 *non-carriers.

Genotyping data from public domains (HapMap and dbSNP) show that *IL-6 *and *ApoE *SNPs show geographic variations (Table 3). MAFs of three *IL-6 *SNPs (rs1800796, rs2069837, and rs1524107) are similar for controls of this Chinese population (0.19-0.27) and CHB of the HapMap project (0.17-0.29). In comparison with these two Chinese populations, for the same *IL-6 *SNPs, Japanese populations have lower MAFs (0.10-0.18), and the MAFs are even lower in Caucasians (0.04-0.08). For *ApoE *SNPs, the MAF of *AopE*112 (rs429358) is highest among Caucasians (dbSNP: 0.15), followed by controls in this Chinese population (0.08), and by Japanese (0.01) and CHB (0.00) from the HapMap Project. The inconsistency between the two Chinese populations for *ApoE*112 may be attributable to the small number of samples genotyped in HapMap study (n = 45 for CHB and n = 43 for JPN). For *ApoE*158 (rs7412), the MAFs were similar between Chinese (0.08-0.11) and Caucasians (0.08) across ethnic groups with a lower value observed in Japanese (0.05).

Hypertension significantly modified the association of *IL-6 *SNPs with LOAD risk. The protective effect of three *IL-6 *SNPs for LOAD was especially evident among participants with hypertension (Table 6), which may be a result of medication for treating hypertension that can lower inflammatory responses (Figure 2). For example, angiotensin receptor blockers or angiotensin-converting enzyme inhibitors have been used to lower blood pressure. Their accompanying neuroprotective effects can reduce neuronal damage and lead to slowed progression of AD [32,33]. Sequence variants of *IL-6 *may lower blood pressure, and subsequent brain hypoperfusion and neural degeneration, which eventually decreases LOAD risk (Figure 2). However, experimental studies are needed to clarify the underlying mechanism.

This study had some strengths. First, the htSNPs in *IL-6 *were identified to explore LOAD risk for the first time. Second, the selection of two representative htSNPs captured abundant genetic information regarding the *IL-6 *gene (r^2 ^= 1.00) as compared to the genetic information captured by the single promoter SNP (rs1800796, r^2 ^= 0.81) in previous candidate-gene studies [12,13]. Third, the associations between *IL-6 *SNPs and LOAD risk remained significant after correction for type I error using FDR, which indicates that these findings are not chance findings. In addition, high false positive rate in genome-wide association studies prevent identifying SNPs associated with disease outcome but with moderate *p *values in the exploratory stage [34]. This study, which selected htSNPs that are representative for Chinese and captured abundant genetic information for *IL-6*, may solve the above issue. Furthermore, brain imaging was used to exclude other diseases with similar presentation as LOAD.

Our study had some limitations. A self-report questionnaire was used to collect information on vascular risk factors (e.g., hypertension, hyperlipidemia, and type 2 DM). Because these diseases/conditions are major health issues, participants' recall of disease/condition diagnosis and their awareness of these diseases/conditions tend to be relatively accurate [35-37]. Therefore, information bias should not be a concern.

## Conclusions

In addition to a significant association of rs1800796 with LOAD, this study for the first time found that an *IL-6 *htSNP (rs1524107) and a haplotype CAT were significantly associated with LOAD risk after correction for multiple tests. These associations remained significant in *ApoE e4 *non-carriers only. Because the majority of AD patients are *ApoE e4 *non-carriers (61% in this study and another Chinese study [13]), *IL-6 *SNPs may be important markers in predicting LOAD risk in *ApoE e4 *non-carriers. In addition, hypertension significantly modified the association of *IL-6 *polymorphisms with LOAD risk. Future large studies are warranted to explore the role of *IL-6 *in risk of LOAD.

## Abbreviations

Aβ: Beta amyloid; AD: Alzheimer's disease; AOR: Adjusted odds ratio; ApoE: Apolipoprotein; CHB: Han Chinese in Beijing China; CI: Confidence interval; CRP: C-reactive protein; DM: Diabetes mellitus; FDR: False discovery rate; HAP: Haplotype; htSNPs: Haplotype-tagging SNPs; HWE: Hardy-Weinberg equilibrium; LD: Linkage disequilibrium; LOAD: Late-onset Alzheimer's disease; MAF: Minor allele frequency; SNP: Single nucleotide polymorphism.

## Competing interests

The authors declare that they have no competing interests.

## Authors' contributions

SYC: data analysis and manuscript writing; TFC: participant recruitment; LCL: technical review; JHC: participant recruitment and study design; YS: participant recruitment; LLW: biospecimen collection and treatment; PKY: participant recruitment; YMC: biospecimen collection and treatment; YCC: conceive of this study, genetic data analyses, manuscript writing. All authors of this research paper have directly participated in the planning, execution, or analysis of the study. All authors of this paper have read and approved the final version submitted.
